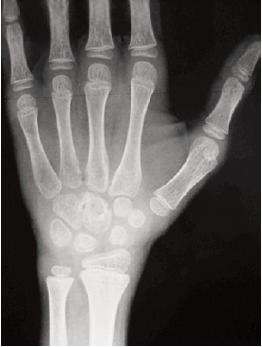# Tuberculosis of Capitate Bone in A Skeletally Immature Patient: A Case Report

**DOI:** 10.5704/MOJ.1403.005

**Published:** 2014-03

**Authors:** J Prakash

**Affiliations:** Department of Orthopaedics, Lady Hardinge Medical College , New Delhi, India

## Abstract

Primary Tuberculosis of wrist joint and carpal bone is rare, and when occurring it usually affects adults. We present a case of isolated tuberculosis of capitate bone; which was confirmed with intra-osseous tissue histopathological examination in a skeletally immature 12-year old boy. There was no signs of reactivation seen at follow-up 18 months after treatment.

## Introduction

Tuberculous bacilli have lived in symbiosis with mankind since time immemorial. Articular tuberculosis accounts for 1-3% of all tuberculosis cases and wrist joint is one of the few rare sites. Tuberculosis of the wrist joint is more common in adults. The disease starts in the synovium and rapidly disseminates to the whole carpus and frequently involves the flexor or extensor tendons1. We present tuberculous involvement of the capitate bone in a skeletally immature person, which has not thusfar been reported to our knowledge.

## CASE REPORT

**Fig. 1: Clinical photograph at presentation showing sinus on dorsal aspect with no redness of skin.**
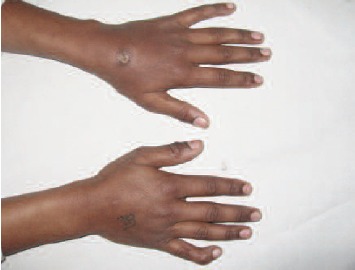


We present a case of a 12 year old male who presented with swelling and a non-healing sinus in the dorsum of the left wrist joint of 5 months’ duration.(Figure 1) The patient gave a history of intermittent low grade fever associated with significant weight loss (5kg) over the last 5 months. There was a history of purulent discharge from the sinus. Patient was on broad spectrum antibiotics at presentation.

Examination revealed the wrist mildly swollen and tender but not warm. A single sinus was present in the dorsum of the wrist. Systemic examination including the respiratory system was essentially normal.

The blood invesigations revealed anemia (haemoglobin-9.2 mg/dl), elevated E.S.R of 52 mm/hr and a Mantoux test of 22mm by 22mm. C- reactive protein was positive (17 mg/l).

Radiograph of the wrist at presentation suggested osteopenia of carpal bones with a lytic lesion of the capitate . Other carpal boness were relatively spared.(Figure 2a) and (Figure 2b) MRI showed increased signal intensity in the capitate with surrounding soft tissue edema in T2 weighted images.(Figure 3) suggesting an infective etiology

Histopathology of intra-osseous tissue removed at biopsy confirmed the diagnosis of bone tuberculosis, with typical caseous necrosis surrounded by epithelioid and giant-cell follicles. Synovial biopsy revealed non-specific chronic synovitis. No organisms were cultured in the purulent material.

The patient was started on multidrug chemotherapy isoniazid 5mg/kg, rifampicin 10mg/kg, pyrazinamide 25mg/kg and ethambutol 15mg/kg. The antitubercular drug was continued for one year. Pyrazinamide was stopped after 3 months and ethambutol at 6 months. The sinus healed in 10 weeks. The wrist became non tender and regained excellent range of motion. Subsequent radiographs showed remineralisation of bone with increasing sclerosis. At 18 months follow up there was no sign of reactivation.

## Discussion

Musculoskeletal tuberculosis is a chronic and progressive disease that mostly affects weight bearing joints^2^. Upper extremity presentations are not common^3^ and diagnosis may be late with an unusual clinical picture.

Wrist joint tuberculosis mostly presents as painless, progressive swelling through tendons mostly without any other systemic sign or evidence of tuberculosis^1,5^. However our case presented with painful progressive swelling with no involvement of tendons, along with constitutional symptoms.

Diagnosis is confirmed on histology. A negative pus culture or inability to see acid fast bacilli under microscope does not exclude tuberculosis^5^. Surgical debridement is controversial for wrist joint tuberculosis^1,5^. Any surgical debridement must be planned after a period of chemotherapy as pre-surgical chemotherapy prevents bony destruction and dissemination of disease^1^. Multidrug chemotherapy must be continued for 9 or 12 months.

The case report presents number of unique features. In terms of age, wrist involvement is rare in children. Early biopsy in our case avoided undue delay in diagnosis that is often seen in wrist tuberculosis. Also isolated carpal involvement is unusual as the disease generally advances rapidly to involve the wrist. High index of suspicion of tuberculosis should prevail when dealing with chronic lesions of the wrist joint. Early diagnosis not only improves the outcome but also prevents surgery and subsequent wrist joint arthrosis.

**Fig. 2a: AP radiograph at presentation showing a lytic lesion in capitate.**
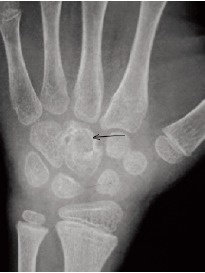


**Fig. 2b: Lateral radiographs of wrist at presentation showing a lytic area in capitate.**
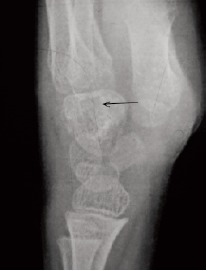


**Fig. 3: T2 weighted image of MRI showing hyperintense lesion in capitate along with soft tissue synovitis.**
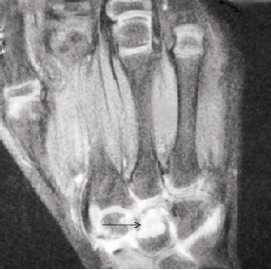


**Fig. 4: Plain radiograph at 9 month follow up showing remineralisation and sclerosis of lesion.**